# Fitness of B-Cell Responses to SARS-CoV-2 WT and Variants Up to One Year After Mild COVID-19 – A Comprehensive Analysis

**DOI:** 10.3389/fimmu.2022.841009

**Published:** 2022-05-02

**Authors:** Benjamin Meyer, Paola Andrea Martinez-Murillo, Barbara Lemaitre, Géraldine Blanchard-Rohner, Arnaud M. Didierlaurent, Paola Fontannaz, Chloé Eugercios Manzanas, Paul-Henri Lambert, Natasha Loevy, Laurent Kaiser, Julie Sartoretti, Chantal Tougne, Jean Villard, Angela Huttner, Claire-Anne Siegrist, Christiane S. Eberhardt

**Affiliations:** ^1^Center for Vaccinology and Neonatal Immunology, Department of Pathology and Immunology, University of Geneva, Geneva, Switzerland; ^2^Division of Laboratory Medicine, Department of Diagnostics and of Medical Specialties, Geneva University Hospitals and University of Geneva, Geneva, Switzerland; ^3^Pediatric Immunology and Vaccinology Unit, Division of General Pediatrics, Department of Pediatrics, Gynecology and Obstetrics, Geneva University Hospitals and University of Geneva, Geneva, Switzerland; ^4^Pediatric Platform for Clinical Research, Department of Woman, Child and Adolescent Medicine, Geneva University Hospitals and Faculty of Medicine, University of Geneva, Geneva, Switzerland; ^5^Division of Infectious Diseases, Geneva University Hospitals, Geneva, Switzerland; ^6^Laboratory of Virology, Division of Laboratory Medicine, Geneva University Hospitals, Geneva, Switzerland; ^7^Geneva Centre for Emerging Viral Diseases, Geneva University Hospitals, Geneva, Switzerland; ^8^Division of General Pediatrics, Department of Woman, Child and Adolescent Medicine, Faculty of Medicine, University of Geneva, Geneva, Switzerland; ^9^Immunology and Transplant Unit, Division of Nephology and Hypertension, Geneva University Hospital and Faculty, Geneva, Switzerland; ^10^Center for Clinical Research, Geneva University Hospitals and Faculty of Medicine, University of Geneva, Geneva, Switzerland; ^11^Center for Vaccinology, Geneva University Hospitals, Geneva, Switzerland

**Keywords:** SARS-CoV-2, plasmablasts, memory B cells, antibody response, COVID-19, neutralization, avidity

## Abstract

**Objective:**

To comprehensively evaluate SARS-CoV-2 specific B-cell and antibody responses up to one year after mild COVID-19.

**Methods:**

In 31 mildly symptomatic COVID-19 participants SARS-CoV-2-specific plasmablasts and antigen-specific memory B cells were measured by ELISpot. Binding antibodies directed against the proteins spike (S), domain S1, and nucleocapsid (N) were estimated using rIFA, ELISA, and commercially available assays, and avidity measured using thiocyanate washout. Neutralizing antibodies against variants of concern were measured using a surrogate-neutralization test.

**Results:**

Plasmablast responses were assessed in all participants who gave sequential samples during the first two weeks after infection; they preceded the rise in antibodies and correlated with antibody titers measured at one month. S1 and N protein-specific IgG memory B-cell responses remained stable during the first year, whereas S1-specific IgA memory B-cell responses declined after 6 months. Antibody titers waned over time, whilst potent affinity maturation was observed for anti-RBD antibodies. Neutralizing antibodies against wild-type (WT) and variants decayed during the first 6 months but titers significantly increased for Alpha, Gamma and Delta between 6 months and one year. Therefore, near-similar titers were observed for WT and Alpha after one year, and only slightly lower antibody levels for the Delta variant compared to WT. Anti-RBD antibody responses correlated with the neutralizing antibody titers at all time points, however the predicted titers were 3-fold lower at one year compared to one month.

**Conclusion:**

In mild COVID-19, stable levels of SARS-CoV-2 specific memory B cells and antibodies neutralizing current variants of concern are observed up to one year post infection. Care should be taken when predicting neutralizing titers using commercial assays that measure binding antibodies.

## Introduction

Tremendous efforts have been undertaken to understand immune responses to SARS-CoV-2 infection and vaccination. The identification of immune correlates of protection is critical in determining whether and when vaccination should occur after infection. The immunogenic SARS-CoV-2 spike (S) protein contains the receptor-binding domain (RBD) which allows for host cell entry, and several vaccine manufacturers have adopted the S-protein from the initial SARS-CoV-2 strain as antigen. Population-based models studying rates of protection therefore use anti-S or anti-RBD antibody titers to predict protection after vaccination (1). However, various SARS-CoV-2 variants have since emerged. Structural changes to their spike proteins were shown to impact antibody neutralization capacity (2–4) substantially more than T-cell recognition (5–7). Neutralizing antibodies are thought to play an important role in the prevention of infection after vaccination in immunocompetent individuals (1, 8). Their persistence and breadth have been followed for 8 months after vaccination and waning of neutralizing antibodies depends on the vaccine platforms (9).

Nevertheless, broad access to COVID-19 vaccination remains limited chiefly to high-income countries, whereas population immunity in low- and middle-income countries increases mainly through infections (10). Therefore, it is of particular interest to determine the durability of B-cell responses after mild infection, for both antibody titers and memory B cells (MBCs). The latter are crucial upon re-exposition, such as reinfection or vaccination of previously infected individuals, when only low antibody titers remain. Within days following the second encounter, MBCs differentiate into cells secreting high-affinity antibodies. Consequently, previously SARS-CoV-2-infected patients require only a single vaccine dose to reach similar or higher antibody titers than immunologically naive individuals after 2 doses (11). Hence long-term persistence of SARS-CoV-2 specific MBCs could potentially spare vaccine doses in countries with a high proportion of convalescent individuals. It is also mostly unclear whether infections with ancestral strains (mainly D614G) elicit neutralizing antibodies functional against emerging SARS-CoV-2 variants, and thus whether convalescent individuals awaiting vaccination may remain protected.

Here we analyze the longitudinal kinetics of early and long-term B-cell responses in 31 COVID-19 patients with mild symptoms, with a focus on SARS-CoV-2-specific memory B-cell responses and kinetics of neutralizing antibodies against the ancestral SARS-CoV-2 strain and various variants of concern (VOC).

## Methods

### Study Cohort

**Figure 1:**
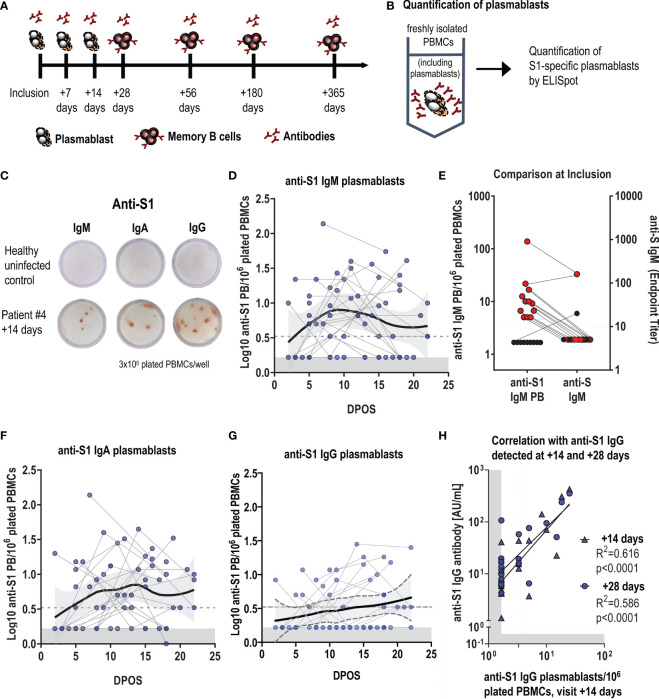
Early plasmablast responses following SARS-CoV-2 infection correlate with antibody titers. **(A)** Schematic of the timepoints following infection when blood samples were collected for the assessment of SARS-CoV-2 specific plasmablast, antibody and memory B-cell responses. **(B)** Experimental design for the quantification of S1-specific plasmablasts (PBs) from fresh blood by ELISpot, with **(C)** representative ELISpot wells of a healthy control (top) and an infected patient (#4) at visit +14 days (bottom). 3x10^5^ fresh PBMCs were plated per well, and each red spot corresponds to one S1-specific IgM, IgA or IgG- secreting plasmablast. **(D, F, G)** Log-transformed number of S1-specific PBs per million plated PBMCs for IgM, IgA and IgG, depending on the days post-onset of symptoms (DPOS). Each dot connected by a line corresponds to one patient and positive data points were fitted using LOESS regression with the 95% confidence interval. Limit of detection are 3,2 PBs per 1 million plated cells, and the grey area indicates levels in healthy controls (n=10). **(E)** shows at the first visit (inclusion) the presence of S1-specific IgM plasmablasts and concomitant measured rIFA anti-S IgM antibody titres in the blood. Results from the same individual are connected with a line. **(H)** Correlation between log-transformed anti-S1 IgG plasmablasts at visit +14 days and log-transformed anti-S1 IgG antibody levels at the same (+14 days) and the following visit (+ 28 days). Dotted line represents the cut-off of the assay, the grey area indicates the detection limit, calculated as 50% of the cut-off of the assay.

This prospective observational monocentric study was conducted at the Geneva University Hospitals (HUG), Switzerland, including participants within 48 hours after positive SARS-CoV-2 RT-qPCR and their subsequently positively tested household contacts. The study was approved by the Geneva Cantonal Ethics Commission (2020-00516) and informed consent was obtained from all participants or their legal representatives. In total, 36 patients were enrolled in the study from March until August 2020, covering the first epidemic wave. Three withdrew after the first visit, one withdrew after the second visit and one patient was finally determined PCR-negative (**Supplementary Figure 1A**). From the remaining 31 RT-qPCR confirmed acute COVID-19 patients we collected longitudinal samples at seven visits at approximately day 0, 7, 14, 28, 56, 180 and 365 post inclusion (**Figure 1A**). For 16 patients we were able to obtain samples at all 7 visits, 25 patients came back around day 180 and 19 around day 365, however 4 had been vaccinated before their final visit (details see **Supplementary Figure 1B**). Seventeen participants were female, 14 male, with an age range from 4-74 years and a median age of 30 years (interquartile range 14.5-38.5 years). Self-reported ethnic groups included mostly Caucasian, followed by African, Hispanic as well as Haitian and mixed (**Table 1**). All cases except one were mild according to WHO classification (12). One participant required a 12-day hospitalization for pneumonia with low-flow oxygen therapy for 8 days, and a second participant was hospitalized for social reasons. Median duration of symptoms was 11 days (**Table 1**). From the same cohort, our group has published a comparative analysis of innate immune responses in children and adults and has used data on memory B cell responses from visit 5, + 56 days, from n=7 children and n=12 adults (11).

**Table 1:** Demographics of included patients.

	No.
**COVID-19 PCR+ Patients (number)**	31
**Gender (numbers)**	
Female	17
Male	14
**Age (years)**	
Min	4
Median (IQR)	30 (14.5-38.5)
Max	74
**Ethnicity (self-reported)**	
African	5
Caucasian	20
Haiti	1
Hispanic	4
Mixed	1
**Duration of acute illness (days)**	
Min	0
Median (IQR)	11 (4.5-17)
Max	58
**Hospitalizations**	
numbers	2/31
**C-reactive Protein at inclusion**	
Individuals with positive CRP levels (> 10 mg/mL)	4/26
Median (IQR), mg/mL	17.7 (159.8)

### B Cell ELISpot Assay

B-cell ELISpot assay was used to evaluate the frequency of plasmablasts and memory B cells as previously described (13, 14). As the detection of plasmablast does not require stimulation, freshly isolated PBMCs were directly plated onto ELISpot plates (**Figure 1B**).

For memory B-cell assays, cryopreserved PBMCs were thawed and stimulated for 6 days with R848 (Sigma, 1µg/mL) and IL-2 (Peprotech, 10 ng/mL) at 37°C, 5% CO_2_.

ELISpot plates were coated overnight at 4°C with S1-protein (Sino biological, #40591-V08H, 100 ng/well), N-protein (1-419) (PROSPEC, # sars-040-c 1mg/mL), anti-human IgG (1 µg/well, Jackson, Ref 709-005-149) or anti-human IgA and IgM (1 µg/well, Jackson, Ref 109-005-064) or as positive control with quadrivalent influenza vaccine (dilution 1/20, Vaxigrip Tetra, season 2019/2020). Plates were blocked with RPMI+FBS 10% (supplemented with 1% Penicillin, Streptomycin and L-Glutamine) and cells were plated in a 3-fold dilution. After six hours of incubation (37°C and 5% CO2), biotinylated human IgG-specific (Jackson ImmunoResearch, Ref 709-066-098), IgA-specific (Jackson 109-065-011), or IgM-specific (Invitrogen, Cat H15015) antibodies were added followed by horseradish peroxidase (HRP)-conjugated Streptavidin (Streptavidin-POD conjugate, Roche, Ref 11089153001) to detect antibody-secreting cells. Plates were developed with 3-Amino-9-ethylcarbazole substrate, wells were acquired (ELISPOT Reader CTL) and spots were manually counted.

### Antibody Characterization by Recombinant Immunofluorescence Assay (rIFA)

To determine IgM antibody titres against the full trimerized S-protein, we used SARS-CoV-2 spike (S) protein-based rIFA as previously described (14). Briefly, pCG1 vector expressing SARS-CoV-2 S-protein (kindly provided by M. Hoffmann and S. Pöhlmann, DPZ Göttingen) was transfected into Vero B4 cells using Fugene HD (Promega #E2311). S-protein expressing cells were spotted on multitest microscopy slides (DUNN Labortechnik GmbH #40-412-05), incubated for 6h and fixed with ice-cold Acetone/Methanol (1:1 mixture). To perform the rIFA, sera were inactivated for 30min at 56°C. To remove IgG antibodies, sera were treated with Eurosorb reagent (Euroimmun AG #1270-0145) according to the manufacturers' protocol. Slides were rehydrated in PBS + 0.1% Tween 20 (PBS-T) for 5min and blocked using 5% milk in PBS-T for 30min at RT. Sera were diluted in PBS + 5% BSA, starting from 1:10 up to 1:1280. 30μL of diluted sera were applied to each spot and incubated for 1h at 37°C. Slides were washed 3x for 1min with PBS-T and 25μL of secondary Alexa488-conjugated goat anti-human-IgM antibody (Jackson ImmunoResearch #109-545-129), diluted 1:200 in PBS, was applied to each spot. Incubation was done at 37°C for 45min, slides were washed 3x for 1min with PBS-T and briefly rinsed with dH_2_O before mounting with glycerol. Slides were examined using a fluorescence microscope and last dilution with a visible signal was recorded as the titer.

### Antibody Characterization by ELISA

IgG and IgA antibodies directed against the S1 domain of the spike protein of SARS-CoV-2 were determined using a commercially available kit (Euroimmun AG, Lübeck, Germany, #EI 2606-9601 G and EI 2606-9601 A) according to the manufacturer’s instructions. To determine quantitative antibody levels, we included a 2-fold dilution series of a reference serum (set at 100 AU/mL) on each plate. If serum samples were outside the linear range, the assay was repeated with a 1:5, 1:10 or 1:50 pre-dilution. Interpolation was done using Graph Pad Prism version 9.2.0 with a sigmoidal 4PL function.

### Antibody Characterization by Roche Elecsys

SARS-CoV-2 anti-RBD or anti-N total Ig antibodies were measured using the Roche Elecsys S and N assay on a Cobas e801 analyzer (Roche Diagnostics Rotkreuz, Switzerland). Anti-RBD antibodies are measured quantitatively and are expressed in U/mL (manufacturers cut-off 0.8 U/mL), whereas anti-N antibodies are measured semi-quantitatively and are expressed as a cut-off index (sample signal/cut-off signal, manufacturers cut-off >1).

### Antibody Avidity Testing

First, anti-RBD antibody titers were measured using a standard ELISA protocol. Briefly, ELISA (Nunc) plates were coated with SARS-CoV-2 RDB (WT strain, provided by EPFL) 1 µg/mL, incubated for 1 hour at 37°C with 2-fold serial diluted sera, washed, and incubated with anti-human IgG peroxidase-conjugated antibody (Jackson, #109-036-098) for 1 hour at 37°C. Plates were revealed using TMB substrate and HCl and read with an ELISA reader (450nm, SoftMax Pro Version 6.2.2). For avidity testing, serum dilutions were calculated to obtain optical densities (OD) of 1.5 and used in duplicates. ELISA was performed as described above and NH_4_SCN was added at different molarities (0, 0.25, 0.5, 1, 2, 3 and 4M). Obtained ODs were converted in log-transformed % of eluded anti-RBD antibodies and the linear relation between molarity and dissociated anti-RBD antibodies was calculated. Results were reported as molarity at which 50% of antibodies were eluded (Avidity Index; AI). Low, moderate, high and very high avidity antibodies were quantified by calculating the % of antibodies that dissociated between 0- 0.5M, 0.5-1M. 1-2M and above 2M, respectively (AviScan).

### Antibody Characterization by Surrogate Neutralization Assay

To determine the neutralizing capacity of patient sera against wild type (WT) and variant SARS-CoV-2 S proteins we used the V-plex COVID-19 ACE2 neutralization kit (MSD, panel 11, #K15458U). Assays were run according to the manufacturers protocol on a MESO QuickPlex SQ 120 reader. For quantification, a monoclonal antibody standard was run on each plate in duplicate (range 0-20μg/mL). Sera were diluted 1:10 and results were expressed in μg/mL. In case the signal of the 1:10 dilution was outside the linear range of the standard curve, additional dilutions of 1:100 and 1:1,000 were run. All sera were tested in duplicate except for the negative control sera which were tested only in single.

### Data Analysis

All antibody titers were log10-transformed. Graphs and statistics were generated using GraphPad Prism version 9.2.0. LOESS smoothing curves were generated using the R software package version 3.6.1. Patient paired data were compared for the different time points using a mixed-effects-model with Geisser-Greenhouse correction or paired t-test. To correct for multiple comparisons, we used the Tukey test implemented in GraphPad Prism. To calculate correlation coefficients we performed simple linear regression analysis. P values ≤ 0.05 were considered significant. We used the following designation of p values in graphs: ns, not significant; **** p ≤ 0.0001; *** p≤ 0.001; ** p≤ 0.01; * p≤ 0.05.

## Results

### Early Plasmablast Responses to Infection

Upon infection, B cells differentiate into plasmablasts, which are the main source of early circulating low-affinity antigen-specific IgM, followed by class-switched IgG and IgA antibodies. We first evaluated the SARS-CoV-2-specific plasmablast response by ELISpot and focused on the immunogenic S1 subunit of the spike protein as antigen (**Figures 1B, C**). S1-specific plasmablasts were undetectable in healthy individuals (n=10, **Figures 1D, F, G**). In infected participants, S1-specific-plasmablast responses were very heterogeneous despite the correction for time post onset of symptoms (POS). S1-specific IgM plasmablasts were highest at around one-week POS (**Figure 1D**). At the inclusion visit, between day 0 and day 9 after symptom-onset, we could already detect SARS-CoV-2-specific IgM plasmablasts in 13 out of 22 participants, while anti-S IgM serum antibodies were not yet measurable (**Figure 1E** and **Supplementary Figure 2A**). S1-specific IgA plasmablasts reached highest levels between week 1 and 2 after symptom onset (**Figure 1F**), followed by anti-S1 IgG plasmablasts (**Figure 1G**). In 22 participants who had three sequential blood drawings over the 3 weeks following symptom-onset, S1-specific IgM, IgA or IgG plasmablasts were detectable at least once. Anti-S1 IgG plasmablast responses measured at the visit +14 days correlated with anti-S1 IgG serum antibody responses at the same visit and 2 weeks later (**Figure 1H**), and antibody titers in the blood followed similar kinetics as plasmablasts (**Supplementary Figures 2A**–**C**).

These data illustrate that an early and rapid antibody response is elicited even in mildly infected individuals, and that the short-lived early plasmablast response contributes to peak responses in antibody levels.

### Long-Term Immune Responses

**Figure 2:**
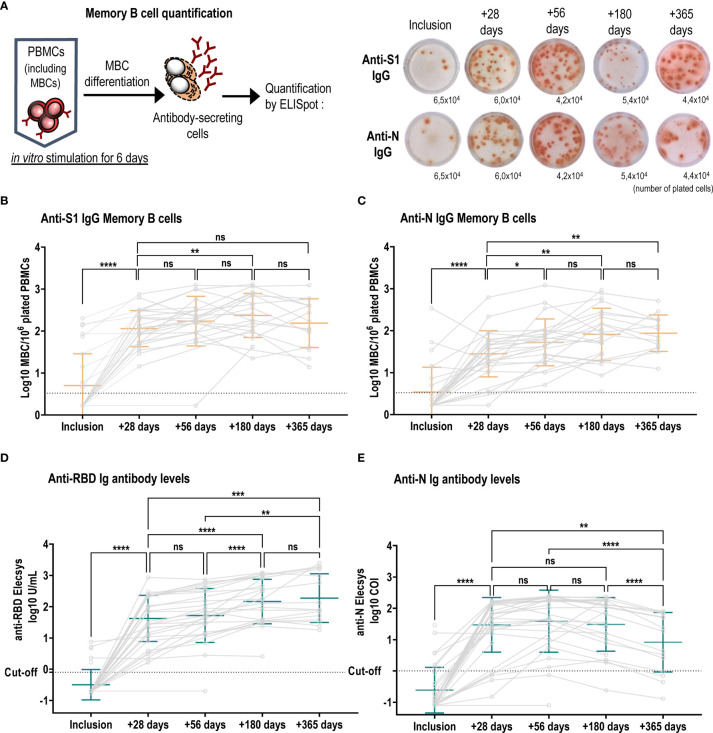
Long-term persistence of SARS-CoV-2-specific memory B cells and antibody responses. **(A)** Left schematic illustrates MBC *in vitro* mitogenic stimulation and differentiation required to become antibody secreting cells. Right, representative wells of anti-S1 IgG and anti-N IgG MBC ELISpot in patient number 9, one spot corresponds to one MBC, and the indicated numbers are stimulated plated cells per well. Numbers of anti-S1-specific **(B)** and anti-N-specific **(C)** IgG MBCs were plotted at each visit and individual patient values are shown in grey circles and connected by grey lines. Anti-S1-specific MBCs increased over time (median ratio at +56 days/inclusion: 52, at +180 days/inclusion: 62.6 and at median ratio +365 days/inclusion: 85.1) and were higher as compared to anti-N specific IgG MBCs (median ratio at +28 days/inclusion: 15; at +56 days/inclusion: 19, at +180 days/inclusion: 29, + 365 days/inclusion: 34). Mean +/- SD is shown in orange. Kinetics of anti-RBD **(D)** and anti-N **(E)** antibody titres over the course of one-year post infection. Individual values are shown in grey circles and patients are connected by grey lines. Mean +/- SD is shown in green. **(D)** anti-RBD Ig antibodies increased over the first 6 months [inclusion *vs* +28 days mean log10 U/mL: -0.50 ± 0.48 *vs* 1.62 ± 0.74, p<0.0001, (+28 days *vs* +180 days mean log10 U/mL: 1.62 ± 0.74 *vs* 2.16 ± 0.72, p<0.0001)], and were stabilized between 6 months and a year (+180 days *vs* +365 days mean log10 U/mL: 2.16 ± 0.72 *vs* 2.27 ± 0.78, p=0.6361), whereas **(E)** anti-N antibodies increased significantly between inclusion and +56 days: mean log10 COI: -0.61 ± 0.73 *vs* 1.58 ± 0.99, p<0.0001) followed by a significant decline up to one year (+56 days *vs* +360 days mean log10 COI: 1.58 *vs* 0.91, p<0.0001). ns, not significant; ****p ≤ 0.0001; ***p ≤ 0.001; **p ≤ 0.01; *p ≤ 0.05.

We next measured SARS-CoV-2-specific memory B-cell responses by ELISpot (**Figure 2A**) in a total of 28 volunteers and studied their kinetics in 13 of them up to 13 months after symptoms onset (+365 days). SARS-CoV-2 infection did not substantially influence the frequency of other antigen-specific MBCs as anti-influenza IgG MBCs and median changes between the four timepoints were less than 2-fold (**Supplementary Figure 2D**). As expected, no SARS-CoV-2-specific MBCs were detectable in pre-pandemic samples of five control individuals.

Most patients elicited anti-S1 IgG MBC responses as early as one month after inclusion (median ratio +28 days/inclusion: 30.4). Anti-S1 IgG MBCs significantly increased up to 6 months (n=22) and remained stable at one year (n=14) (**Figure 2B**). All 26 tested patients also developed anti-S1 IgA MBCs (**Supplementary Figure 2E**), albeit at lower frequencies (median ratio at +28 days/inclusion: 3.5; at +56 days/inclusion: 4.3; at +180 days/inclusion: 18). We assessed the kinetics of MBCs against the full-length nucleocapsid, a protein that is partially conserved between betacoronaviruses. We found similar kinetics compared to the S1-protein with a sustained response after 2 months, but the magnitude of the response was reduced compared to S1-specific MBCs (**Figure 2C**).

To evaluate the long-term antibody response after mild SARS-CoV-2 infection, we used Roche Elecsys S (anti-RBD) and N assays, which are the standard assays available in many clinical laboratories measuring SARS-CoV-2-specific total Ig levels. Anti-RBD antibody titers increased between the inclusion visit and one-month post infection. Subsequently, we observed a significant rise in anti-RBD titers between one and 6 months, after which titers remained stable (**Figure 2D**). In contrast, anti-N antibodies reached a peak at two months post infection but significantly declined between two months and one year (**Figure 2E**).

**Figure 3:**
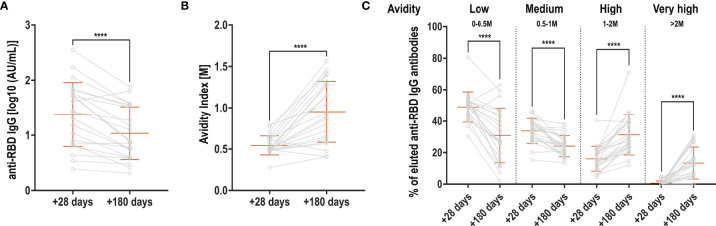
Evolution of RBD-specific antibody avidity. **(A)** Anti-RBD antibodies at +28 and +180 days, expressed in log10-transformed AU/mL **(B)** Avidity index: molarity of thiocyanate for which 50% of the antibodies dissociated at +28 and +180 days **(C)** Aviscan: proportion of antibodies with low (0-0.5M), medium (0.5-1M), high (1-2M) and very high (>2M) avidity at +28 and +180 days. Orange bars represent mean + SD. Light grey lines connect samples from the same patient (grey circles). Two-sided paired t-test **(A + B)** or Wilcoxon matched-pairs signed rank test **(C)** were used to calculate significance for comparisons between timepoints ****p < 0.0001.

We next asked if antibody affinity maturation over time could explain the sustained signal in the Roche Elecsys S (anti-RBD) assay. We selected 22 patients for whom sequential serum samples were available at +28 days/inclusion and at +180 days/inclusion and anti-RBD IgG titers significantly decreased when quantified in an in-house ELISA (**Figure 3A**). Antibody avidity was measured using thiocyanate at different molarities, and represented as avidity index, indicating the thiocyanate molarity at which 50% of anti-RBD IgG antibodies dissociated. We observed a significant increase in avidity over the 5 months (**Figure 3B**). To better discriminate the antibody avidity, we performed Aviscan, which allows to establish a profile of antibodies with low, medium, high and very high avidity. At +28 days/inclusion, antibody avidity was mostly low or medium, and very little very-high avidity antibodies were detectable (**Figure 3C**). At +180 days/inclusion, antibodies with low and medium avidity significantly decreased in favor of a significant rise in antibodies with high and very high avidity.

Together, these data demonstrate that mild COVID-19 infections induce long-lasting IgG S1 and N-specific memory B cells up to 13 months post infection, and whilst antibody titers decrease, RBD-specific antibodies undergo potent affinity maturation, potentially affecting some commercial assays measuring antibody binding.

### Neutralizing Antibody Responses to WT and Variants of Concern

**Figure 4:**
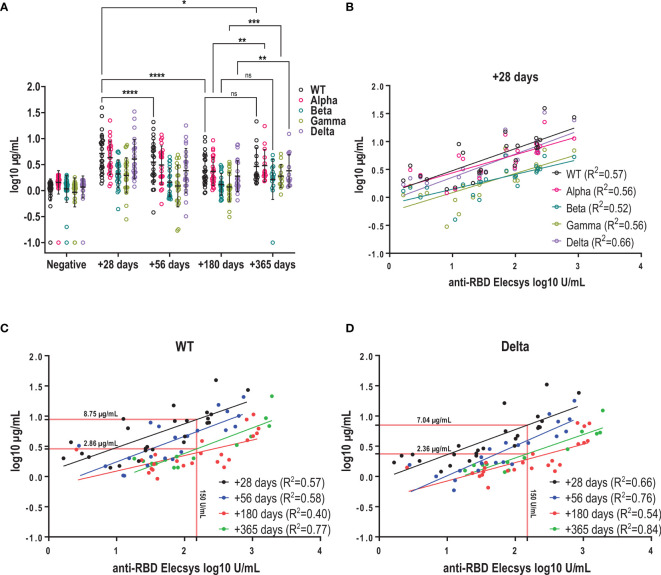
Kinetics of neutralizing antibodies against the infectious strain and variants of concern. **(A)** Kinetics of neutralizing antibody titres of WT and VOCs using a surrogate RBD-ACE2 binding inhibition assay. Mean +/- SD is shown in black. Antibodies with neutralizing capacity against the WT were stable between 6 and 12 months (mean as log (µg/mL) +180 days *vs* +365 days: 0.38 ± 0.3 *vs* 0.46 ± 0.36) **(B)** Correlation between log-transformed anti-RBD and neutralizing antibody titers against SARS-CoV-2 WT and variants using linear regression. **(C + D)** Correlation between anti-RBD and neutralizing antibody titres for WT and Delta variant at the different time points post infection. Red lines show interpolated neutralizing titre at an anti-RBD titres of 150U/mL. ns, not significant; ****p < 0.0001; ***p < 0.001; **p < 0.01; *p < 0.1.

Over the observation period, four new SARS-CoV-2 variants of concern (VOC) have emerged as designated by WHO (15). We asked how a primary infection with the wild type (WT) would influence the antibody recognition and especially their functionality in terms of neutralization capacity against the Alpha, Beta, Gamma and Delta variants. We showed that neutralizing capacity against the WT peaked at one-month post-infection and declined until 6 months (mean log 10 µg/mL 0.71 ± 0.39 *vs* 0.38 ± 0.3, p<0.0001) after which it remained stable up to one year (**Figure 4A**). Similar kinetics were observed for VOCs up to 6 months albeit at reduced titers. At +28 days, mean neutralizing antibody titers against the Alpha and Delta variant were around 20% lower compared to the WT (**Figure 4A** and **Supplementary Figure 3A**), whereas for the Beta and Gamma variant a more pronounced decrease in the neutralizing capacity was observed (around 60% lower, **Figure 4A** and **Supplementary Figure 3A**). Between 6 months and one year we observed a significant increase in neutralizing antibody titers for the Alpha (mean log10 µg/mL: 0.37 ± 0.26 *vs* 0.48 ± 0.31, p=0.008), Gamma (mean log10 µg/mL: 0.07 ± 0.28 *vs* 0.28 ± 0.22, p=0.0002) and Delta variant (mean log10 µg/mL: 0.28 ± 0.3 *vs* 0.38 ± 0.31, p=0.0089), but not for the Beta variant (mean log10 µg/mL: 0.12 ± 0.21 *vs* 0.2 ± 0.39, p=0.4554) in the entire cohort (**Figure 4A**). When only samples were analyzed for which both the 6-month and one-year timepoint were available, we also detected an increase in neutralizing titers for the WT and Beta variant in addition to all other variants (**Supplementary Figure 3B**).

Furthermore, we observed that the decline in neutralizing antibody titers was more prominent for the WT compared to the VOC over the one-year period, resulting in near-similar titers for WT and Alpha at one year, and only around 30% lower titers for Beta and Gamma compared to 20% and 60% at +28 days, respectively. Kinetics of decay of neutralizing antibodies against Delta were similar to the one against WT and we measured titers that were 15% lower at one year compared to the WT (**Supplementary Figure 3A**).

In clinical practice, antibody responses to SARS-CoV-2 infection or vaccination are mainly determined by standard methods such as the Elecsys S assay rather than using surrogate neutralization assays. Therefore, we asked how well RBD titers predict the neutralizing response for the WT and VOCs over time. We found a moderate to good correlation between RBD titers and neutralizing antibodies for VOCs with correlation coefficients (R^2^) ranging between 0.52 and 0.66 at one month (**Figure 4B**). Overall, for both WT and Delta, anti-RBD titers showed a moderate to good correlation with neutralizing antibody titers for all time points with R^2^ ranging between 0.4-0.77 for the WT and 0.54-0.84 for the Delta variant (**Figures 4C, D**). However, over the course of one year we observed that neutralizing titers decreased but corresponding anti-RBD levels remained stable: This means that a RBD level of 150 U/mL measured at one-month post symptom onset would predict a neutralizing antibody titer of 8.75 µg/ml (WT) or 7.04 µg/mL (Delta), whereas the same RBD level measured at one-year post infection would predict approximately 3-fold lower neutralizing titers (WT: 2.86 µg/mL; Delta: 2.36 µg/mL, **Figure 4C, D**). In summary, mild SARS-CoV-2 infection elicited antibodies capable of neutralizing VOCs at lower potency in addition to the infectious strain. Antibodies were sustained up to one year after infection, but binding antibodies may overestimate the neutralizing capacity when measured several months after infection.

## Discussion

This longitudinal study provides evidence that even mild COVID-19 infection can induce a potent early plasmablast response, persistent SARS-CoV-2-specific memory B-cell and sustained antibody responses of increasing avidity and with neutralizing capacity of the infectious strain and VOC after one year.

After SARS-CoV-2 infection, plasmablasts rapidly secrete anti-S1 SARS-CoV-2 IgM antibodies, followed by germinal center-derived class-switched anti-S IgG or IgA antibodies. Responses were heterogenous despite correction for time since onset of symptoms, which could be due to individual differences in the incubation period and thus timing of plasmablast peak responses. Circulating plasmablasts contribute to the initial peak antibody response, observed in our and other cohorts between 2 and 3 weeks (16), which then stabilizes when only plasma cells persist in the bone marrow as reported by others (17). These SARS-CoV-2 specific bone-marrow resident plasma cells have a quiescent phenotype when detected 7 months after infection (17), and most likely contribute to the persistent antibody responses observed after 12 months in our cohort.

The germinal center reaction also elicits memory B cells, and in most participants SARS-CoV-2-specific MBCs induced at the time of infection persisted over a year, as also described by others (18, 19). The recall potential of MBCs is witnessed by higher S-protein-specific plasmablast levels detected after second vaccine doses (8) as compared to the primary responses seen in our cohort, and by a steep rise in antibody titers observed shortly after reinfection with SARS-CoV-2 (20). Also, vaccine-specific antibody titers are higher after a single dose of mRNA vaccine given to convalescent as compared to naïve individuals (11). In addition, MBCs elicited after naïve or convalescent vaccination have the potential to secrete neutralizing antibodies (21). Although our participants had only mild disease, MBCs follow comparable kinetics in severe COVID-19 patients (22). This knowledge can help to spare vaccine doses in countries with high rates of SARS-CoV-2 infection and low vaccine supply when there is proof of pre-infection and in absence of highly mutated variants.

The breadth of the memory B-cell repertoire was shown to increase with time after infection. It has been speculated that persistent antigen could increase the rate of somatic hypermutations and affinity maturation (23, 24). This potentially applies to other germinal center outputs, such as plasmablasts and plasma cells, as sustained germinal center reaction in lymph nodes have been observed after COVID-19 vaccination (8).

This antigen persistence could partly explain our observation that anti-RBD antibody titers measured with the commercialized Roche Elecsys S assay did not decrease over time. This is in line with other reports using the same method (25, 26) or traditional ELISA (27), the latter showing no substantial reduction in anti-Spike or anti-RBD IgG antibodies over 8 months. However, there are studies which report a decrease in RBD-specific antibody titers over the course of one year (24, 28, 29). Herein measured anti-N and neutralizing antibody titers, using a surrogate assay detecting the inhibition of binding between RBD and ACE-2, followed the expected kinetics similar to other studies that report a shorter half-life for anti-N antibodies (27), with a decline for up to one year (29–31).

We showed that RBD-specific antibody avidity increased over time, favored by the potential antigen persistence after infection, and to which the Roche Elecsys S assay measuring anti-RBD antibodies is possibly sensitive to. A similar phenomenon has been observed for yellow fever vaccinations where antibody affinities increase over time while neutralizing titers declined (32). Interestingly, we also observed a significant rise in neutralizing titers against the Alpha, Gamma and Delta variant between 6 months and one year, which was also present for the WT and the Beta variant when only paired samples were analyzed. It is conceivable that the observed increase in neutralizing capacity against VOCs is the result of affinity maturation. In agreement with our findings, another study revealed an increase in antibody affinity leading to higher neutralization capacity and increased resistance to viral escape mutants (33). Whether the antibody affinity maturation induced through vaccination, especially after the 3^rd^ dose, will lead to a similar phenomenon seems plausible, but is currently unknown.

Neutralizing antibody titers have been shown on a population basis to correlate with protection from symptomatic infection (1). Neutralizing antibodies are difficult to measure, and most clinical laboratories use antibody-binding assays to determine the level of immunity in vaccinated or recovered individuals. We could show that anti-RBD titers measured with the Roche assay one month after symptom onset predict the neutralizing capacity for WT and VOCs well, albeit at different levels. Similar results were obtained in other studies (34, 35). However, we found that anti-RBD titers predicted 3-fold lower absolute neutralizing antibodies at one year compared to one month for the WT and Delta variant. Therefore, we urge that the time of infection and vaccination be considered when interpreting anti-RBD titers and trying to predict neutralizing capacity of antibodies.

In conclusion, mild COVID-19 infection elicits neutralizing antibodies specific to the infectious strain and current VOCs, and durable memory B-cell responses that are readily recallable, suggesting potential protection against reinfection in those awaiting vaccination.

## Data Availability Statement

The datasets presented in this article are not readily available because no consent has been obtained from participants for public sharing of anonymized datasets. Requests to access the datasets should be directed to CE, christiane.eberhardt@unige.ch.

## Ethics Statement

The studies involving human participants were reviewed and approved by Geneva Cantonal Ethics Commission (2020-00516). Written informed consent to participate in this study was provided by the participants’ legal guardian/next of kin.

## Author Contributions

Conception of study: C-AS, CE, AD, AH, LK; Protocol: C-AS, CE, AD, AH; Patient recruitment: NL, GB-R, AH; Experiments and analysis and interpretation of data: BM, PM, BL, LK, CM, JS, JV, P-HL, GB-R; Manuscript writing: BM, PM, CE; Review of manuscript: all authors. All authors contributed to the article and approved the submitted version.

## Funding

This work was supported by the Swiss National Science Foundation (grant number 31CA30_196732/1 to C-AS).

## Conflict of Interest

The authors declare that the research was conducted in the absence of any commercial or financial relationships that could be construed as a potential conflict of interest.

## Publisher’s Note

All claims expressed in this article are solely those of the authors and do not necessarily represent those of their affiliated organizations, or those of the publisher, the editors and the reviewers. Any product that may be evaluated in this article, or claim that may be made by its manufacturer, is not guaranteed or endorsed by the publisher.

## Supplementary Material

Supplementary Figure 1 Summary of enrolled patients, per-protocol timing and accomplished study visits.

Supplementary Figure 2 Kinetics of early antibody responses and Memory B cells. Graphs illustrating the kinetics of antibody responses following SARS-CoV-2 infection with **(A)** anti-S IgM antibody titers measured by rIFA and anti-S1 IgG **(B)** and IgA **(C)** antibody titers using a commercially available kit. Each dot connected by a line corresponds to one patient and data points were fitted using LOESS regression with the 95% confidence interval. Numbers of antigen-specific MBCs were plotted at each visit and individual patient values are shown in gray circles and connected by gray lines. Mean +/- SD is shown in orange, and the dotted line indicates the limit of detection. Graph in **(D)** summarizes Influenza-specific IgG MBCs, and fold-changes between visits were minimal: median: 1.4 for both, ratio +28 days/inclusion and +56 days/inclusion; median: 1.2 + 180 days/inclusion, median: 1.4 + 365 days/inclusion. The graph in **(E)** shows anti-S1-specific IgA MBCs.

Supplementary Figure 3 Antibody responses to VOC during mild COVID-19. **(A)** Fold change of VOCs compared to the WT strain of SARS-CoV-2 at different time points post infection. Mean +/- SD is shown in black. **(B)** Comparison of neutralizing antibody titres of all available paired serum samples at 6 months and one-year post onset of symptoms for SARS-CoV-2 WT and variants. Paired samples, i.e. from the same patient are connected with black lines.
