# Supplementary material for: Fitness of B-Cell Responses to SARS-CoV-2 WT and Variants Up to One Year After Mild COVID-19 – A Comprehensive Analysis
Source: Front Immunol. 2022 May 2;13:841009. doi: 10.3389/fimmu.2022.841009 (PMC9108245; doi:10.3389/fimmu.2022.841009)
Supplement: Supplementary Figure 1 — Summary of enrolled patients, per-protocol timing and accomplished study visits. [file DataSheet_1.pdf]

Suppl Fig 1

A

Study flow

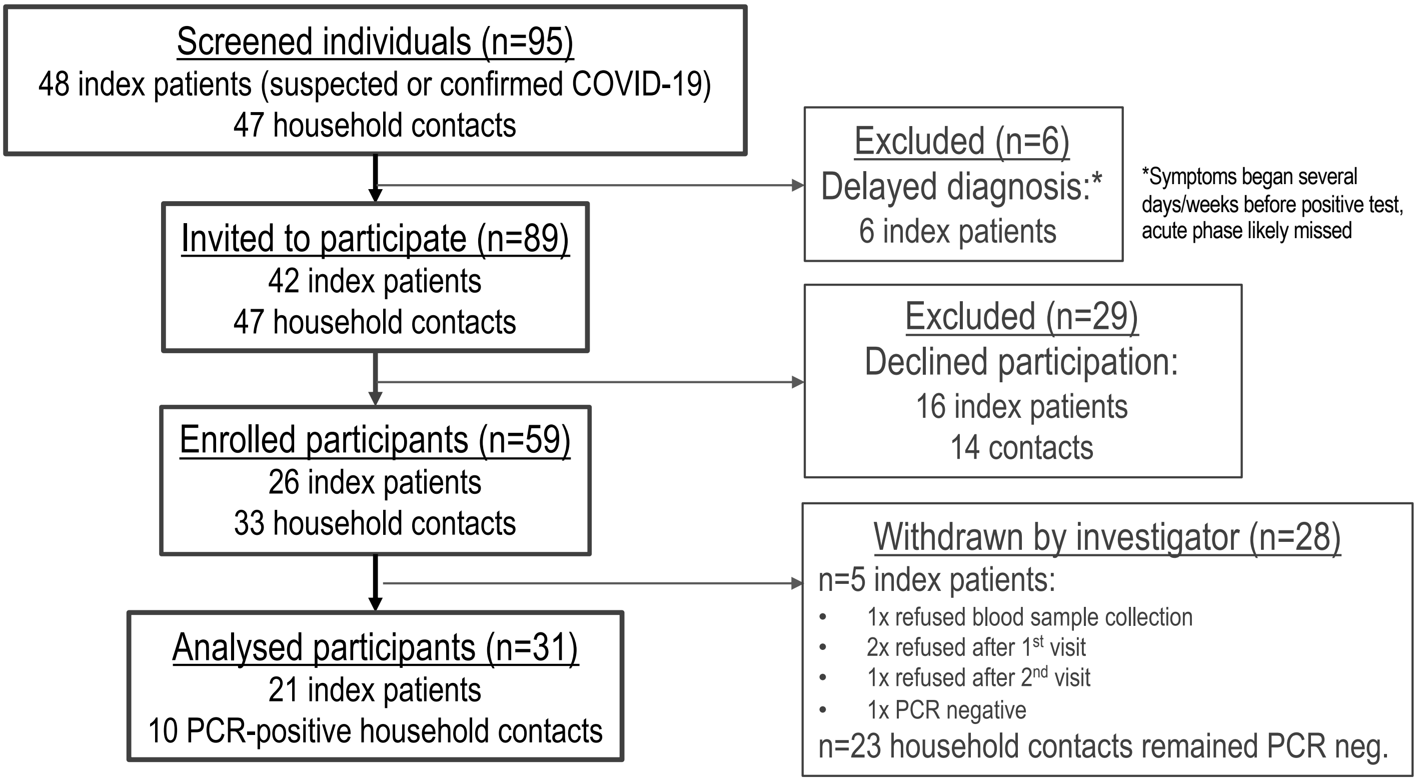

B

Attended study visits

Study visit	Participants remaining in the study	Attended visit
Inclusion visit	31	31
+7 days (+/-2 days)	31	27
+14 days (+/- 3 days)	31	25
+28 days (+/- 7 days)	29	26
+56 days (+/- 14 days)	28	22
+180 days (+/- 30 days)	25	25
+365 days (+/- 45 days)	19 *	19

*(n=15 with no missed visit, n=4 vaccinated before visit)

Suppl Fig 2

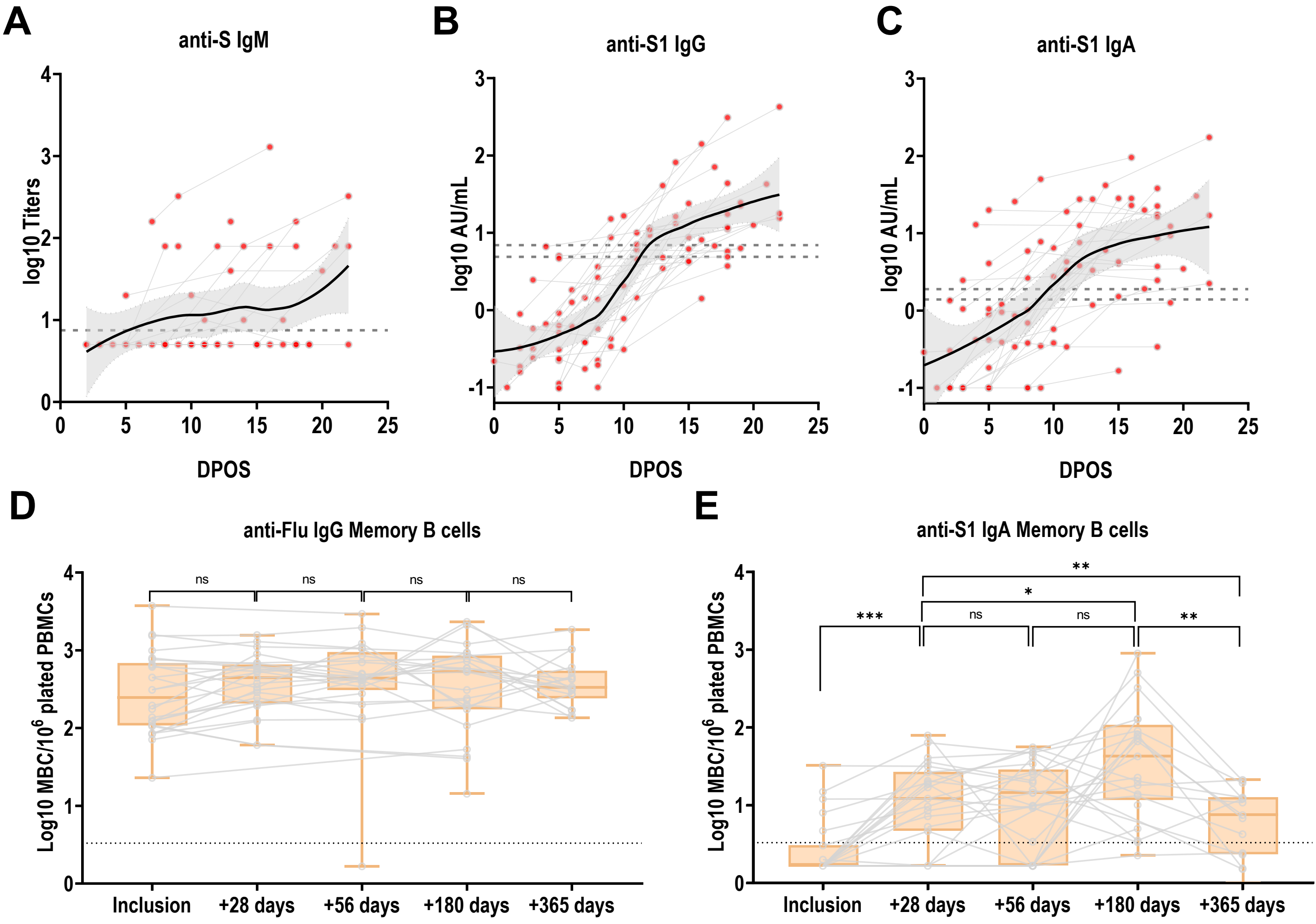
A
anti-S IgM
log10 Titers
DPOS
B
anti-S1 IgG
log10 AU/mL
DPOS
C
anti-S1 IgA
log10 AU/mL
DPOS
D
anti-Flu IgG Memory B cells
Log10 MBC/10^6 plated PBMCs
ns
ns
ns
ns
Inclusion
+28 days
+56 days
+180 days
+365 days
E
anti-S1 IgA Memory B cells
Log10 MBC/10^6 plated PBMCs
**
*
***
ns
ns
**
Inclusion
+28 days
+56 days
+180 days
+365 days

Suppl Fig 3

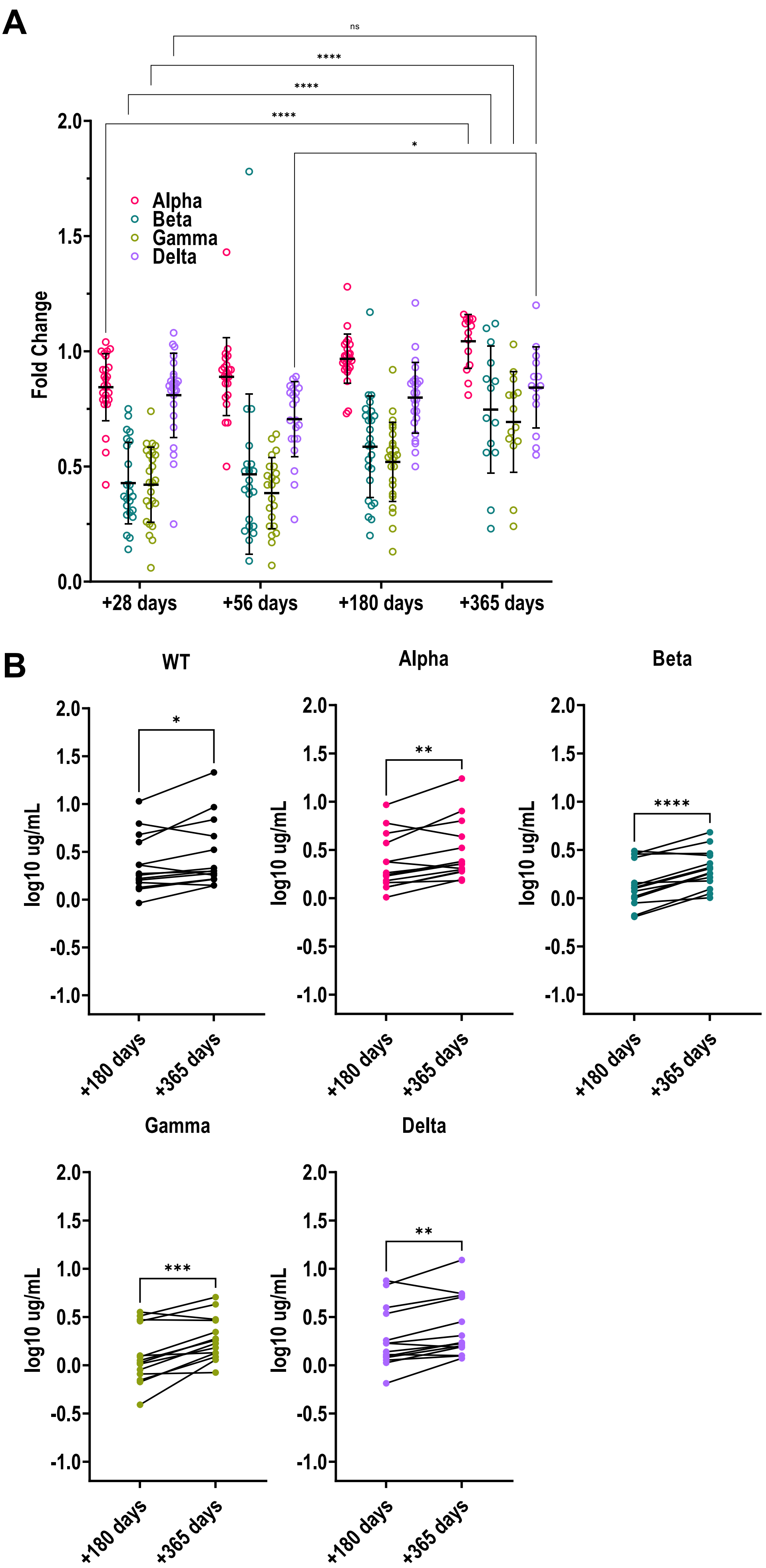
A
ns
****
****
****
*
2.0
1.5
1.0
0.5
0.0
Fold Change
Alpha
Beta
Gamma
Delta
+28 days
+56 days
+180 days
+365 days
B
WT
Alpha
Beta
Gamma
Delta
log10 ug/mL
*
**
****
***
**
2.0
1.5
1.0
0.5
0.0
-0.5
-1.0
+180 days
+365 days
